# Physiological Responses and Stroke Variables during Arm Stroke Swimming Using Critical Stroke Rate in Competitive Swimmers

**DOI:** 10.3390/sports10040046

**Published:** 2022-03-22

**Authors:** Yuki Funai, Masaru Matsunami, Shoichiro Taba, Shigehiro Takahashi

**Affiliations:** 1 Department of Life Wellness, Faculty of Social Welfare, Kumamoto Gakuen University, Kumamoto 862-8680, Japan; 2 Department of Health and Sports, Faculty of Health and Welfare Human Services, St. Catherine University, Matsuyama 799-2496, Japan; matsunami-m@catherine.ac.jp; 3 Department of Sports Science, Faculty of Sports and Health Science, Fukuoka University, Fukuoka 814-0180, Japan; taba@fukuoka-u.ac.jp; 4 Department of Sport Science, School of Health and Sport Sciences, Chukyo University, Toyota 470-0393, Japan; shigehirot0321@gmail.com

**Keywords:** interval training, upper limb, blood lactate concentration, stroke rate, stroke length

## Abstract

The current study examined the physiological responses and stroke variables at critical stroke rate (CSR), 105% CSR, and 110% CSR in order to utilize CSR for prescription arm stroke swimming. Nine male national-level collegiate swimmers performed an all-out 200 m and 400 m for determining the CSR. Participants performed three sets of 6 × 100 m (with 10 s of rest between each bout), the stroke rate for each set was enforced at CSR, 105% CSR, and 110% CSR. Mean swimming velocity, heart rate, and rate of perceived exertion were found to increase with each set (*p* < 0.05). Blood lactate concentration did not differ between the CSR and the 105% CSR (3.3 ± 1.4 vs. 3.5 ± 1.5 mmol/L) but was higher in 110% CSR (5.1 ± 1.6 mmol/L) than in the other two sets (*p* < 0.05). There was no difference in the stroke rate between all bouts in each set, and the stroke length did not change from the second to sixth bout in each set. This study suggested that training intensity for CSR and 105% CSR correspond to threshold level, and 110% CSR corresponds to high-intensity training level. It was also suggested that training in the CSR–110% CSR range could be performed without regard to SL reduction.

## 1. Introduction

Training for competitive swimming includes continuous training, in which swimmers continue to swim without rest intervals. Conversely, there is interval training (IT), in which swimmers repeat multiple bouts with intermittent rest periods. IT is the primary method used in competitive swimming training due to the option to change a combination of components such as swimming velocity, swimming distance, number of repetitions, and rest time.

In competitive swimming training, whole-body swimming, arm stroke swimming, and leg kick swimming improve metabolic capacity and swimming technique [1]. In front crawl swimming, previous studies have reported that the upper limbs contribution to performance of more than 10% [2,3]. It has been reported that the contribution of the upper limbs increases with increasing swimming distance [3]. Therefore, arm stroke swimming training is essential in enhancing swim performance, especially in long-distance front crawl swimmers.

In recent years, training methods that control stroke rate (SR), the number of strokes per minute in front crawl whole-body swimming, have been investigated [4,5]. The critical stroke rate (CSR) has been used as a criterion for SR and is calculated as the slope of the regression line between the total effort swimming time and the total number of strokes [4]. CSR is theoretically defined as the maximum SR that does not cause exhaustion [4]. It was shown that in continuous training using CSR in whole-body swimming, blood lactate concentration was 3.73 ± 0.96 mmol/L, and stroke length (SL) did not decrease throughout the training phase [4]. Therefore, it was considered that using the CSR as a training variable enabled control of training intensity, and could be utilized for technical training [4,6,7].

Funai et al. [8] examined the swimming velocity, physiological responses, and stroke variables in 400 m IT using CSR in arm stroke swimming. The results showed that the swimming velocity and physiological responses did not differ from critical swimming velocity (CV) (the maximum swimming velocity at which lactate production does not exceed oxidative utilization in 400 m IT) [9,10,11,12]. It was also found that the SL decreased during IT at CV, but remained constant during IT at CSR. These results suggest that CSR is an effective training intensity index for IT in arm stroke swimming and that IT at CSR can reduce the loss of propulsive force per stroke.

CSR has been used only in IT for a relatively long distance of 400 m (Long-IT). Additionally, the physiological responses and stroke variables have not been validated for IT repeated for a short distance (Short-IT). Short-IT is frequently introduced in the training of competitive swimmers because the short swimming distance allows them to set a higher swimming velocity than Long-IT. Short-IT also has the advantage of making it easier to increase training intensity [13]. Dalamitros et al., (2016) [14] found that IT at high intensity for 50 m and 100 m effectively improved aerobic capacity in competitive swimmers. Piatrikova et al., (2020) [15] used CV and CSR as criteria for high-intensity training prescription and reported that controlling SR as well as swimming velocity can increase training effectiveness. However, in training with controlled swimming speed, when Long-IT and Short-IT are performed at the same swimming velocity, the physiological exercise intensity is lower during Short-IT [16]. This suggests that the physiological exercise intensity may also be reduced when CSR is used for Short-IT. Therefore, further validation of training intensity settings is needed for CSR to be used in Short-IT.

The purpose of this study was to examine physiological responses and stroke variables at CSR, 105% CSR, and 110% CSR in order to utilize CSR for prescription of Short-IT with arm stroke swimming. It was the hypothesized that the intensity at which the blood lactate concentration begins to accumulate in Short-IT is higher than CSR, and controlling SR will reduce the decrease in SL not only CSR, but also in 105% CSR and 110% CSR.

## 2. Materials and Methods

### 2.1. Participants

Nine well-trained, national-level, male middle- and long-distance swimmers (400 m and 1500 m as a specialty event, respectively) participated in this study (mean ± SD; age: 19.7 ± 1.0 years, height: 1.72 ± 0.04 m, and body mass: 67.7 ± 7.7 kg). The sample size was calculated using G*Power version 3. 1. 9. 7 (*f* = 0.33; *α* = 0.05; 1–*β* = 0.80). The points of participants on the Fédération Internationale de Natation (FINA) scale for each specialty event in a 50 m pool was 754.6 ± 23.8. All swimmers had at least 9 years’ experience as competitive swimmers, and trained 9 times per week for approximately two hours per session, covering a distance of 6000–8000 m per session. Swimmers were briefed on the benefits and risks of the tests before participation, and written informed consents were collected from all participants. The protocol was approved by the Kumamoto Gakuen University ethics review committee (approved date: 16 October 2017). Participants were asked to arrive at the swimming pool in a rested and fully hydrated state, and to abstain from smoking, alcohol, caffein, and strenuous exercise in the 48 h before testing.

### 2.2. Testing Procedure

All tests were performed in a 50 m indoor swimming pool (27.0 ± 0.3 °C water temperature) and involved front crawl arm stroke swimming initiated with a push-off start. All tests were preceded by a standardized warm-up and a 20 min rest period. The standardized warm up based on Neiva et al., (2014) [17] consisted of 200 m whole-body swimming (low–moderate intensity), 2 × 100 m leg kick swimming (moderate intensity) 4 × 50 m (25 m drill/25 m low intensity), 6 × 50 m arm stroke swimming (25 m race pace/25 m low intensity), and 100 m whole-body swimming (low intensity). Arm stroke swimming was performed similarly to the general training method used for competitive swimming. In addition, lower limb muscle activity was restricted by placing training buoys between the legs. The participants were familiar with the training buoys and could swim without moving their lower limbs. All tests were conducted within seven days.

### 2.3. Determination of CSR and CV

For determining CSR and CV, 200 m and 400 m maximal swimming were performed in random order, with two maximal swimming separated by an interval of 24 h. The CSR was considered the slope of the linear regression between the time and the number of stroke cycles expressed as cycles per minute (cycles × min^–1^) according to Franken et al. [5]. The 200 m and 400 m maximal swimming bouts were recorded above the water using an HDR-CX470 digital movie camera operating at 60 Hz (Sony, Japan). The digital movie camera was set up 20 m away from the swimmers so that the entire pool was in the camera field. The recorded videos were analyzed using video analysis software (OTL-8PZ, Octal, Japan) to calculate the swimming time and the number of stroke cycles. When analyzing the swimming time, the lap times for every 50 m were also measured. The mean stroke time was determined from as many strokes as possible between 15 and 35 m in each 50 m. The number of stroke cycles was calculated as the lap time divided by the mean stroke time. One stroke cycle was defined as the unit from the entry of one hand to the following entry of the same hand. As per Wakayoshi et al. [12], the CV was determined as the slope of the linear regression between the swim distance and time.

### 2.4. Interval Test

The participants swam three sets of 6 × 100 m (with 10 s of rest between each bout). The stroke rate for each set was enforced at CSR, 105% CSR, and 110% CSR (set@CSR, set@105% CSR and set@110% CSR, respectively), and the rest interval between each set was 60 s. No set swimming velocity was imposed during the test, but the stroke rate was controlled by a metronome (Tempo Trainer Pro, Finis, USA) placed in the swimmer’s cap. The metronome weighed 19.5 g and had a 4.8 cm width and 1.4 cm thickness. Participants were instructed to swim as fast as possible in a set SR, as this method can easily reduce swimming velocity. Each stroke was considered as the entry of the right or left fingertip to the entry of the next fingertip on the same side, and the strokes were performed in time with the electronic sound.

During the tests, swimmers assessed their heart rate (HR) using HR sensor (H10, Polar, Finland) and rated perceived exertion (RPE) using the Japanese version of the Borg scale [18]. Blood was sampled (3 μL) from the fingertip after each set, and a portable lactate analyzer (Lactate Pro2, ARKRAY, Japan) was used to estimate the blood lactate concentration (BLa). HR, RPE, and BLa were measured immediately after the last bout in each set, and these were representative values for each set.

For verification of swimming velocity, swimming time, and stroke variables in each bout, the interval test was recorded using a digital movie camera from the same position as in the recording of the 200 and 400 m maximal swimming bouts. The recorded videos were analyzed using video analysis software (OTL-8PZ, Octal, Japan). The swimming velocity in each bout was calculated from the swimming time, and the mean swimming velocity for each set was also calculated. The mean stroke time was recorded using the same method as the 200 and 400 m maximal swimming bouts. The SR and SL were calculated by the following equation based on Craig and Pendergast (1979) [19], and expressed as the average of the first and second half.

SR = 60/mean stroke time; SL = swimming velocity × 60/SR.

### 2.5. Statistical Analyses

All results were presented as mean ± standard deviation (mean ± SD). All data were checked for normal distribution using the Shapiro–Wilk test. One-way ANOVA for repeated measures was used to examine differences between each set. The mean swimming velocity, for HR, RPE, and BLa. Two-way ANOVA for repeated measures with the set and bouts was used to analyze differences in swimming velocity, SR, and SL. Mauchly’s test was carried out and Greenhouse–Geisser correlation was applied if sphericity was violated. When a significant interaction was found, a simple main effect test was performed for each level of the factor, and when no interaction was found, the main effect test was performed. Eta squared (*η*^2^) was used to calculate effect size for main effect, *η*^2^ was considered small if the absolute value was between 0.01 and 0.06, medium if it was between 0.06 and 0.14, and large if it was greater than 0.14. When the main effect was found, multiple comparisons Bonferroni test was used to estimate the statistical significance. Cohen’s d (*d*) was used to calculate the effect size for multiple comparisons, *d* was considered small if the absolute value was between 0.2 and 0.5, medium if it was between 0.5 and 0.8, and large if it was greater than 0.8. A significance level of *α* = 0.05 was assumed. All statistical analyses were conducted using BellCurve for Excel software (Version 2.15, Social Survey Research Information Co., Ltd., Tokyo, Japan).

## 3. Results

The mean swimming time in the 200 m and 400 m maximal swimming sessions were 129.71 ± 2.36 s and 272.94 ± 6.15 s, respectively. The mean calculated CSR and CV values were 33.75 ± 3.72 cycles/min and 1.40 ± 0.05 m/s, respectively.

The mean swimming velocity at set@CSR, set@105% CSR, and set@110% CSR in the interval test was 1.39 ± 0.05 m/s, 1.42 ± 0.04 m/s, and 1.45 ± 0.03 m/s, respectively. The HR were 147.89 ± 11.87 bpm, 158.67 ± 14.20 bpm, 163.78 ± 13.65 bpm, and RPE were 12.44 ± 2.13, 13.78 ± 2.05, 15.33 ± 2.00, respectively. A statistically significant main effect (*p* < 0.05) was found between sets related to mean swimming velocity (*p* < 0.001, *F* = 27.969, *η*^2^ = 0.25), HR (*p* < 0.001, *F* = 41.961, *η*^2^ = 0.22), and RPE (*p* < 0.001, *F* = 59.765, *η*^2^ = 0.27). Multiple comparison tests showed that set@105% CSR was significantly higher than set@CSR for each measure (mean swimming velocity: *p* < 0.001, *d* = 0.65; HR: *p* < 0.001, *d* = 0.82; RPE: *p* < 0.001, *d* = 0.64), and set@110% CSR was significantly higher than set@CSR (mean swimming velocity: *p* < 0.001, *d* = 1.29; HR: *p* < 0.001, *d* = 1.24; RPE: *p* < 0.001, *d* = 1.40) and set@105% CSR (mean swimming velocity: *p* < 0.001, *d* = 0.71; HR: *p* < 0.001, *d* = 0.37; RPE: *p* < 0.001, *d* = 0.77) for each measure. The BLa was 3.3 ± 1.4 mmol/L, 3.5 ± 1.5 mmol/L, and 5.1 ± 1.6 mmol/L, and there was a statistically significant main effect between the sets (*p* = 0.001, *F* = 6.310, *η*^2^ = 0.12). Multiple comparison tests showed that set@110% CSR was significantly higher than set@CSR (*p* = 0.014, *d* = 1.21) and set@105% CSR (*p* = 0.036, *d* = 1.07).

Table 1 shows the swimming velocity, swimming time, SR, and SL for each bout in the interval test. The swimming time was also presented in addition to swimming velocity, as coaches are likely to instruct more on swimming time than on swimming velocity. Since there was no statistically significant interaction between set and bout as a factor for all of these measures (*p* > 0.05), we conducted a main effect test for each factor. The results showed a statistically significant main effect between the sets for swimming velocity (*p* < 0.001, *F* = 27.969, *η*^2^ = 0.23), swimming time (*p* < 0.001, *F* = 25.862, *η*^2^ = 0.24), SR (*p* < 0.001, *F* = 183.898, *η*^2^ = 0.13), and SL (*p* < 0.001, *F* = 65.442, *η*^2^ = 0.02). Multiple comparison tests showed that set@105% CSR was significantly higher than set@CSR in all bouts for swimming velocity (*p* < 0.001, *d* > 0.62) and SR (*p* < 0.001, *d* > 0.29), and set@110% CSR was significantly higher than set@CSR (swimming velocity: *p* < 0.001, *d* > 1.06; SR: *p* < 0.001, *d* > 0.77) and set@105% CSR (swimming velocity: *p* < 0.001, *d* > 0.72; SR: *p* < 0.001, *d* > 0.43) in all bouts. Swimming time and SL were significantly shorter in all bouts for set@105% CSR than for set@CSR (swimming time: *p* < 0.001, *d* > 0.64; SL: *p* < 0.001, *d* > 0.29), and for set@110% CSR than for set@CSR (swimming time: *p* < 0.001, *d* > 1.06; SL: *p* < 0.001, *d* > 0.53) and set@105% CSR (swimming time: *p* < 0.001, *d* > 0.46; SL: *p* < 0.001, *d* > 0.33). There was no statistically significant main effect of SR between bouts (*p* = 0.200). On the other hand, there was a statistically significant main effect of swimming velocity (*p* < 0.001, *F* = 18.516, *η*^2^ = 0.02), swimming time (*p* < 0.001, *F* = 18.844, *η*^2^ = 0.02), and SL (*p* = 0.001, *F* = 7.665, *η*^2^ = 0.01) between bouts. Multiple comparison tests showed that swimming velocity and SL were significantly higher in the first bout than the other bouts in all sets (swimming velocity: *p* < 0.001, *d* > 0.28; SL: *p* < 0.014, *d* > 0.26). The swimming time was significantly longer in the first bout than the other bouts in all sets (*p* < 0.001, *d* > 0.27). However, there were no statistically significant differences in the swimming velocity, swimming time, and SL among the second and sixth bout in all sets (*p* > 0.05).

## 4. Discussion

In front crawl whole-body swimming, the main role of lower limb muscle activity is to increase the stability of the lower limb and to maintain the underwater posture [20]. The contribution of lower limb muscle activity to propulsive force is reported to be about 10% [3]. Therefore, most of the propulsive force in the front crawl is contributed by the upper limbs, and the contribution increases as the distance of swimming increases [3]. Arm stroke swimming is an important training method for improving metabolic capacity and stroke technique, especially in long-distance competitive swimming such as 1500 m and open water swimming. In fact, Konstantaki et al., (2008) [21] reported that training that incorporated more arm stroke swimming improved metabolic capacity during upper limb muscle activity and performance in arm stroke swimming. It has been shown that there is a high correlation (r = 0.90) between the endurance performance of arm stroke swimming and whole-body swimming in long-distance swimmers [22].

Funai et al. [8] reported that BLa in Long-IT with CSR was 3.16 ± 1.43 mmol/L in arm stroke swimming. Furthermore, it was found that the swimming velocity with CSR corresponds to CV, which is the maximum swimming velocity at which the production of lactate in Long-IT does not exceed oxidative utilization. Namely, CSR can be used as an indicator of training intensity in Long-IT as well as CV. They also reported that Long-IT with CSR can reduce SL decline, training with CSR may be beneficial as it may contribute to improved upper limb stroke technique. Therefore, validation of physiological exercise intensity and stroke variables during Short-IT in arm stroke swimming with intensity set using CSR could provide useful information to swimming coaches.

In this study, SR was increased by 5% based on CSR in Short-IT, and the mean swimming velocity, HR, and RPE increased with the set (*p* < 0.05). On the other hand, there was no statistically significant difference in BLa between set@CSR and set@105% CSR (*p* = 0.999). However, set@110% CSR was higher than set@CSR and set@105% CSR (*p* = 0.014 and *p* = 0.036, respectively). The breakdown of glycogen produces lactate, and BLa is determined by the balance between lactate production and oxidative utilization [23]. Additionally, the intensity at which BLa increases rapidly indicates a sudden increase in glycogen utilization [23]. In competitive swimmers, the maximum value at which lactate production does not exceed oxidative utilization is considered to be 3.0–3.5 mmol/L [11,12,24,25]. In the present study, not only the set@CSR but also the set@105% CSR BLa corresponded to this range. These results suggest that glycogen utilization increased rapidly between set@105% CSR and set@110% CSR in the present interval test, and lactate production reached an intensity that exceeded oxidative utilization.

Hellard et al. [16] reported that oxygen uptake and BLa were lower in the 100 m × 30 bouts of IT than in the 500 m × six bouts at the Lactate Threshold (LT) swimming velocity in whole-body swimming. In addition, Shimoyama and Nomura [26] reported that BLa was lower in the 100 m × 16 bouts than in the 200 m × eight bouts at the Onset Blood Lactate Accumulation (OBLA) swimming velocity. In IT, it has been previously reported that oxygen uptake during rest periods does not decrease drastically, resulting in enhanced oxidative utilization of lactate [27]. Short-IT is thought to be less prone to lactic acid accumulation than Long-IT due to the greater number of rests in Short-IT. Thus, it has been shown that the physiological exercise intensity decreases when the swimming velocity used in Long-IT is used in Short-IT, and it is thought that the same thing happened in this study in which the intensity was set using CSR. However, there is one point to consider. It has been noted that arm stroke swimming has lower metabolic characteristics than whole-body swimming, as oxygen uptake is about 20% lower and blood lactate concentrations are less to accumulate [1,28]. Therefore, whether there is a difference in physiological exercise intensity between Long-IT and Short-IT in arm stroke swimming needs to be reexamined.

For competitive swimmers, intensity setting according to training objectives is an essential factor affecting training effectiveness [29]. Based on the results of this study, it was considered that CSR cannot be used as a standard SR for categorizing training intensity in Short-IT of arm stroke swimming in the same way as Long-IT. In addition, it should be noted that not only CSR, but 105% CSR also does not reach the intensity at which lactate production exceeds oxidative utilization, and 110% CSR exceeds that intensity. Hence, the intensity should be set according to the purpose of training.

The SR in this study was set at CSR, 105% CSR, and 110% CSR. As a result, SL in each bout decreased significantly with each set (*p* < 0.01, Table 1), and swimming velocity increased with each set (*p* < 0.01, Table 1). These changes in SR, SL, and swimming velocity were the same as the results of studies in which swimming velocity increased training intensity [30,31], suggesting that it is possible to increase training intensity by SR.

When stroke parameters were compared between bouts, SR was considered constant with no significant difference (*p* > 0.05) between bouts in all sets due to the use of a metronome. According to the relationship of swimming velocity = SR × SL, the swimming velocity when SR is controlled is determined by SL. In this study, only SR was controlled, but the swimmers were instructed to swim as fast as possible at the set SR. In this situation, SL was significantly lower (*p* < 0.01) in all the sets after the second bout compared to the first bout. A previous study [30] reported that the SL immediately after the start of training was the longest in the training of competitive swimmers, regardless of their swimming velocity. Therefore, in the present study, the SL of the first bout was the highest not only in set@CSR but also in set@105% CSR and set@110% CSR due to the 60 s rest between sets.

SL was significantly higher in the first bout in all sets, but there was no statistically significant difference between bouts from the second to sixth bout (*p* > 0.05). When the swimming velocity was controlled in Long-IT, it was reported that BLa was 3.77 ± 1.52 mmol/L, and SL was decreased [8]. For swimming training in which velocity was controlled, it was found that the decrease in SL was more pronounced at higher BLa values [30,31]. A potential reason for the reduction in SL when swimming velocity is controlled is a decrease in the temporal proportion of the propulsive pull and push phases among the four phases that comprise the upper limb stroke (glide-catch phase, pull phase, push phase, and recovery phase). It has also been reported that this decrease in proportion is linked by an increase in lactate production [32,33].

On the other hand, it was reported that BLa in Long-IT with SR controlled by CSR was 3.16 ± 1.43 mmol/L, and its swimming velocity did not differ from CV, but SL was not decreased [8]. In the present study, BLa at 110% CSR was 5.1 ± 1.6 mmol/L, and SL did not change after the second bout as in CSR and 105% CSR, even though lactate production was considered to exceed oxidative utilization. Alberty et al. [32] analyzed SR-controlled maximal swimming and found that the temporal proportions of the four phases of the upper limb stroke did not change between the beginning and end of maximal swimming. Therefore, it is considered that controlling the SR in this study also did not reduce the SL because the temporal proportion of the four phases of the upper limb stroke did not change from the second to the sixth bout in all sets.

In competitive swimmers, training at maximal or higher intensity, where lactate production does not exceed oxidative utilization, can effectively improve metabolic function by increasing oxygen uptake, lactate utilization, and muscle buffering capacity [9]. However, when such intensity training is performed with controlled swimming velocity, the higher the training intensity, the more likely it is that stroke technique will be impaired. Therefore, the fact that SL did not decrease after the second bout in all sets of set@CSR, set@105% CSR, and set@110% was considered to be a training that could solve the problem of conventional training with controlled swimming velocity.

It has been pointed out that monitoring SR is important to evaluate stroke technique during training in conventional training methods with controlled swimming velocity [34], and SR and SL are used as objective stroke data for coaching. On the other hand, this study suggested that training could be conducted with less SL decline by controlling SR. This point is considered to be the usefulness of controlling SR. Therefore, it is suggested that arm stroke swimming training based on CSR can be utilized as training for the upper limbs in the front crawl.

Although the above findings were obtained in this study, there are several limitations. First, it cannot be denied that the fact that SL was constant from the second to the sixth bout in all sets may be the result of the verification with Short-IT. This point can be clarified by comparing the results with those of Short-IT in which the swimming velocity was controlled. Second, although the number of bouts used in this study was six in each set, the number of bouts may be higher in the field of instruction. Therefore, the results of this study may be limited to this protocol, and it is necessary to verify the physiological responses and stroke variables by changing the components of IT in the future. Third, this study was conducted using arm stroke swimming, a local training method in competitive swimming. In the future, it might be necessary to apply CSR to whole-body swimming and to verify its effectiveness as a training method to enhance performance.

## 5. Conclusions

This study suggested that training intensity for CSR and 105% CSR correspond to threshold level, and 110% CSR corresponds to high-intensity training level. It was also suggested that training in the CSR–110% CSR range could be performed without regard to SL reduction.

## Figures and Tables

**Table 1:** Changes in the swimming velocity, swimming time, and stroke variables for each bout corresponding at CSR, 105% CSR, and 110% CSR during the interval test.

		First	Second	Third	Fourth	Fifth	Sixth
Swimming velocity (m/s)	CSR	1.41 ± 0.05	1.39 ± 0.05 ^1^	1.39 ± 0.06 ^1^	1.38 ± 0.05 ^1^	1.38 ± 0.05 ^1^	1.38 ± 0.05 ^1^
	105% CSR	1.44 ± 0.04 ^a^	1.42 ± 0.04 ^a,1^	1.42 ± 0.04 ^a,1^	1.41 ± 0.04 ^a,1^	1.42 ± 0.04 ^a,1^	1.42 ± 0.04 ^a,1^
	110% CSR	1.46 ± 0.04 ^a,b^	1.45 ± 0.04 ^a,b,1^	1.44 ± 0.03 ^a,b,1^	1.44 ± 0.03 ^a,b,1^	1.44 ± 0.04 ^a,b,1^	1.45 ± 0.03 ^a,b,1^
Swimming time (s)	CSR	71.13 ± 2.47	71.98 ± 2.73 ^1^	72.09 ± 2.80 ^1^	72.41 ± 2.73 ^1^	72.41 ± 2.82 ^1^	72.32 ± 2.52 ^1^
	105% CSR	69.68 ± 2.09 ^a^	70.36 ± 2.19 ^a,1^	70.58 ± 1.88 ^a,1^	70.95 ± 2.06 ^a,1^	70.75 ± 2.10 ^a,1^	70.69 ± 1.83 ^a,1^
	110% CSR	68.73 ± 2.05 ^a,b^	69.23 ± 1.65 ^a,b,1^	69.34 ± 1.47 ^a,b,1^	69.33 ± 1.35 ^a,b,1^	69.34 ± 1.86 ^a,b,1^	69.26 ± 1.60 ^a,b,1^
Stroke rate (cycles/min)	CSR	33.39 ± 3.80	33.69 ± 3.70	33.97 ± 3.87	33.66 ± 3.64	33.70 ± 3.85	33.67 ± 3.44
	105% CSR	35.23 ± 3.78 ^a^	35.34 ± 3.90 ^a^	35.24 ± 3.88 ^a^	35.24 ± 3.73 ^a^	35.41 ± 3.76 ^a^	35.21 ± 3.69 ^a^
	110% CSR	36.84 ± 4.10 ^a,b^	37.00 ± 4.00 ^a,b^	36.89 ± 3.73 ^a,b^	37.17 ± 3.89 ^a,b^	37.16 ± 4.15 ^a,b^	37.17 ± 4.11 ^a,b^
Stroke length (m/cycle)	CSR	2.55 ± 0.22	2.50 ± 0.21 ^1^	2.47 ± 0.21 ^1^	2.48 ± 0.20 ^1^	2.48 ± 0.22 ^1^	2.48 ± 0.20 ^1^
	105% CSR	2.47 ± 0.27 ^a^	2.44 ± 0.22 ^a,1^	2.44 ± 0.23 ^a,1^	2.42 ± 0.22 ^a,1^	2.42 ± 0.21 ^a,1^	2.43 ± 0.21 ^a,1^
	110% CSR	2.39 ± 0.21 ^a,b^	2.37 ± 0.22 ^a,b,1^	2.37 ± 0.21 ^a,b,1^	2.35 ± 0.22 ^a,b,1^	2.35 ± 0.21 ^a,b,1^	2.35 ± 0.22 ^a,b,1^

Data are expressed as the mean ± standard deviation. ^a,b^ Values significantly different to CSR and 105% CSR, respectively; ^1^ Value significantly different to the first bout (*p* < 0.05).

## References

[1] Ogita F., Hara M., Tabata I. (1996). Anaerobic capacity and maximal oxygen uptake during arm stroke, leg kicking and whole body swimming. Acta Physiol. Scand..

[2] Deschodt V.J., Arsac L.M., Rouard A.H. (1999). Relative contribution of arms and legs in humans to propulsion in 25-m sprint front-crawl swimming. Eur. J. Appl. Physiol. Occup. Physiol..

[3] Silveira R.P., de Souza Castro F.A., Figueiredo P., Vilas-Boas J.P., Zamparo P. (2017). The effects of leg kick on swimming speed and arm-stroke efficiency in the front crawl. Int. J. Sports Physiol. Perform..

[4] Dekerle J., Sidney M., Hespel J.M., Pelayo P. (2002). Validity and reliability of critical speed, critical stroke rate, and anaerobic capacity in relation to front crawl swimming performances. Int. J. Sports Med..

[5] Franken M., Diefenthaeler F., Moré F.C., Silveira R.P., de Souza Castro F.A. (2013). Critical stroke rate as a parameter for evaluation in swimming. Mot. Rev. De Educ. Física.

[6] Dekerle J. (2006). The use of critical velocity in swimming: A place for critical stroke rate?. Port. J. Sport. Sci..

[7] Raimundo J.A., Ribeiro G., Lisbôa F.D., Pereira G.S., Loch T., De Aguiar R.A., Martins E.C., Caputo F. (2020). The effects of predictive trials on critical stroke rate and critical swimming speed. J. Sports Med. Phys. Fit..

[8] Funai Y., Matsunami M., Taba S. (2019). Physiological responses and swimming technique during upper limb critical stroke rate training in competitive swimmers. J. Hum. Kinet..

[9] Machado M.V., Júnior O.A., Marques A.C., Colantonio E., Cyrino E.S., De Mello M.T. (2011). Effect of 12 weeks of training on critical velocity and maximal lactate steady state in swimmers. Eur. J. Sport Sci..

[10] Minganti C., Demarie S., Comotto S., Meeusen R., Piacentini M.F. (2012). Evaluation of critical swimming velocity in young amateur swimmers. Sport Sci. Health.

[11] Takahashi S., Wakayoshi K., Hayashi A., Sakaguchi Y., Kitagawa K. (2009). A method for determining critical swimming velocity. Int. J. Sports Med..

[12] Wakayoshi K., Yoshida T., Udo M., Harada T., Moritani T., Mutoh Y., Miyashita M. (1993). Does critical swimming velocity represent exercise intensity at maximal lactate steady state?. Eur. J. Appl. Physiol. Occup. Physiol..

[13] Maglischo E.W. (2003). Swimming Fastest.

[14] Dalamitros A.A., Zafeiridis A.S., Toubekis A.G., Tsalis G.A., Pelarigo J.G., Manou V., Kellis S. (2016). Effects of short-interval and long-interval swimming protocols on performance, aerobic adaptations, and technical parameters: A training study. J. Strength Cond. Res..

[15] Piatrikova E., Willsmer N.J., Sousa A.C., Gonzalez J.T., Williams S. (2020). Individualizing Training in Swimming: Evidence for Utilizing the Critical Speed and Critical Stroke Rate Concepts. Int. J. Sports Physiol. Perform..

[16] Hellard P., Dekerle J., Nesi X., Toussaint J.F., Houel N., Hausswirth C. (2010). Ventilatory and biomechanical response analysis in short vs. long interval training sessions in elite long distance swimmers. Proceedings of the XIth International Symposium for Biomechanics and Medicine in Swimming.

[17] Neiva H.P., Marques M.C., Barbosa T.M., Izquierdo M., Marinho D.A. (2014). Warm-up and performance in competitive swimming. Sports Med..

[18] Onodera K., Miyashita M. (1976). A study on Japanese scale for rating of perceived exertion in endurance exercise (in Japanese). Jpn. J. Phys. Educ..

[19] Craig A.B., Pendergast D.R. (1979). Relationships of stroke rate, distance per stroke, and velocity in competitive swimming. Med. Sci. Sports.

[20] Yanai T. (2001). Rotational effect of buoyancy in frontcrawl: Does it really cause the legs to sink?. J. Biomech..

[21] Konstantaki M., Winter E., Swaine I. (2008). Effects of arms-only swimming training on performance, movement economy, and aerobic power. Int. J. Sports Physiol. Perform..

[22] Ogasawara K., Shimada K., Tachi M., Wakayoshi K. (2009). Determination of critical velocity in arm strokes, leg kicks and whole crawl strokes for competitive swimmers and water polo players (in Japanese). Jpn. J. Sci. Swim. Water Exerc..

[23] Hatta H. (2008). Lactate, an Efficient Energy Source.

[24] Martin L., Whyte G.P. (2000). Comparison of critical swimming velocity and velocity at lactate threshold in elite triathletes. Int. J. Sports Med..

[25] Pelarigo J.G., Denadai B.S., Greco C.C. (2011). Stroke phases responses around maximal lactate steady state in front crawl. J. Sci. Med. Sport.

[26] Shimoyama Y., Nomura T. (1999). Role of rest interval during interval training at OBLA speed. Proceedings of the VIIIth International Symposium for Biomechanics and Medicine in Swimming.

[27] Olbrecht J., Madsen O., Mader A., Liesen H., Hollmann W. (1985). Relationship between swimming velocity and lactic concentration during continuous and intermittent training exercise. Int. J. Sports Med..

[28] Ribeiro J., Figueiredo P., Sousa J., Monteiro J., Pelarigo J., Vilas-Boas J.P., Toussaint H.M., Fernandes R.F. (2015). VO_2_ kinetics and metabolic contributions during full and upper body extreme swimming intensity. Eur. J. Appl. Physiol..

[29] Mujika I., Chatard J.C., Busso T., Geyssant A., Barale F., Lacoste L. (1995). Effects of training on performance in competitive swimming. Can. J. Appl. Physiol..

[30] Keskinen K.L., Komi P.V. (1993). Stroking characteristics of front crawl swimming during exercise. J. Appl. Biomech..

[31] Pelarigo J.G., Greco C.C., Denadai B.S., Fernandes R.J., Vilas-Boas J.P., Pendergast D.R. (2016). Do 5% changes around maximal lactate steady state lead to swimming biophysical modifications?. Hum. Mov. Sci..

[32] Alberty M., Potdevin F., Dekerle J., Pelayo P., Gorce P., Sidney N. (2008). Changes in swimming technique during time to exhaustion at freely chosen and controlled stroke rates. J. Sports Sci..

[33] Bassan N.M., César T.E., Denadai B.S., Greco C.C. (2016). Relationship between fatigue and changes in swim technique during an exhaustive swim exercise. Int. J. Sports Physiol. Perform..

[34] Barden J.M., Kell R.T. (2009). Relationships between stroke parameters and critical swimming speed in a sprint interval training set. J. Sports Sci..

